# Predictors of anti-glycaemic medication-taking among adults with diabetes mellitus seeking care in a tertiary hospital in Cape Coast, Ghana

**DOI:** 10.4314/gmj.v56i3.10

**Published:** 2022-09

**Authors:** Amaris TD Baah, George Adjei, Sebastian Eliason

**Affiliations:** 1 Greater Accra Regional Hospital, Ridge, Accra; 2 Department of Community Medicine, School of Medical Sciences, University of Cape Coast, Cape Coast

**Keywords:** Anti-glycaemic Medication, Diabetes Mellitus, Cape Coast, Teaching Hospital

## Abstract

**Objectives:**

This study sought to assess the level of anti-glycaemic medication-taking and its predictors among adults living with diabetes receiving treatment at Cape Coast Teaching Hospital (CCTH).

**Design:**

This was a cross-sectional study carried out among adults living with diabetes and receiving care at CCTH. Data on socio-demographic characteristics and anti-glycaemic medication-taking were gathered using a structured questionnaire. A scale consisting of 4 domains (filling prescribed medication; taking medications appropriately according to the instructions of healthcare professionals; practising behavioural modifications, and showing up for follow-up appointments) and eight items was used to measure the level of anti-glycaemic medication-taking. Descriptive statistics, chi-square test (and Fisher's exact test where appropriate), bivariate and multivariate logistic regression models were used in analysing the data.

**Setting:**

The study was carried out in the diabetes clinic in Cape Coast Teaching Hospital.

**Participants:**

The total enumerative sampling technique was used to select 250 adults living with diabetes and receiving care at CCTH.

**Main outcome measures:**

Anti-glycaemic medication-taking

**Results:**

Out of 250 participants studied, 42% had high anti-glycaemic medication-taking. Predictors of anti-glycaemic medication-taking included; forgetfulness (aOR=0.02, 95% CI: 0.00–0.64, p<0.001), patient's involvement in treatment plan (aOR=0.12, 95% CI: 0.02–0.64, p=0.014) and having good knowledge about one's medication (aOR=2.34, 95% CI: 1.10–4.98, p=0.028).

**Conclusion:**

Less than half of the sample population (42%) had high anti-glycaemic medication-taking, with forgetfulness, involvement in the treatment plan and good knowledge about anti-glycaemic medications, predicting medication-taking.

**Funding:**

None declared

## Introduction

Diabetes mellitus is a metabolic disorder characterised by chronic hyperglycaemia and derangements in carbohydrate, lipid and protein metabolism.^1^ It is a chronic disease that occurs either when the pancreas does not produce enough insulin or when the body cannot effectively use the insulin it produces. In other words, it occurs as a result of insulin deficiency or impaired effectiveness of insulin's action, or a combination of the two.^1^ Diabetes occurs in both the poor and the affluent. Approximately 77% of the global impact of diabetes on health was recorded in developing countries. Again, about 50% of the patients said to have diabetes fall within the ages of 40 and 59 years.^2^

Diabetes mellitus is a chronic disease with a rapidly rising incidence. Studies show that the worldwide occurrence of diabetes in adults was 9.3% in 2019 and is projected to increase to 11% by 2045.^3^ Also, according to the International Diabetes Federation (IDF), individuals who had diabetes in 2019 were about 463 million, with an expected increase to 578.5 million by 2030. 4

Lessening the risk of long-term diabetes complications by keeping blood sugar levels within the normal range through a healthy diet, exercise, and medication is what is desired.^1^ Without taking medication as prescribed, and implementing recommended lifestyle changes, treatment goals cannot be fully realised in the light of advanced medical technology.

Medication-taking goes beyond just taking medications as ordered by a physician. It is the extent to which a person's behaviour corresponds with agreed recommendations from a health care provider.^5^

Factors that connote medication-taking may include: appropriately taking medication according to the health professional's instructions, practising behavioural modifications, and showing up for follow-up appointments.^5^ Failure to do one or all of the above will suggest a failure of medication-taking and could negatively impact health. A recent study has shown that, people living with diabetes and hypertension, people with high levels of blood lipids or those with biventricular heart failure who did not take their medications according to specified treatment regimens were more than twice at risk of hospitalisation compared with the general population.^6^ Medication-taking can be ascertained at various points in the treatment therapy, including the commencement of treatment, going for refills, taking the correct dose, and taking the medication until the last date.^7^

Medication-taking is important in the management of chronic illnesses, including diabetes. Nevertheless, some individuals do not often take their medications as prescribed. World Health Organization (WHO) reported that approximately 50% of patients with chronic diseases in high-income countries do not take medications as prescribed. In developing countries, where access to medicines and healthcare is inadequate, with inappropriate diagnosis, medication-taking related to chronic illnesses is poorer.^8^ Again, several studies conducted in other countries have reported poor consistency in taking hypoglycaemic drugs among people living with diabetes in such countries. These studies also discussed factors that bring about such poor consistency in taking anti-diabetic drugs.^9^,^10^,^11^,^12^,^13^ The average rate of medication-taking is reported to be between 8% and 90%.^14^ According to one study, an estimated $289 billion is lost every year due to individuals not taking medications as prescribed .^15^ Beyond the increased health costs, it can further lead to failure to realise optimal clinical benefits, thus decreasing the therapeutic expectations of both the physician and patient.

Despite the evidence on anti-glycaemic medication- taking and its associated effects in other countries and Ghana, it was not clear what the level and predictors of anti-glycaemic medication-taking were, among people living with diabetes, receiving care at the Cape Coast Teaching Hospital (CCTH), which is the largest referral tertiary facility serving the Central Region of Ghana.^12^,^16^,^17^ Thus, this study sought to assess the level of medication-taking and its predictors among adults with diabetes mellitus.

## Methods

### Study design

A hospital-based cross-sectional study was conducted among adults living with diabetes who were on anti-glycaemic medication.

### Study area

This study was carried out in the Cape Coast Teaching Hospital (CCTH) in the Central Region of Ghana. It is, a 400-bed hospital situated in the northern part of Cape Coast. The hospital provides general Out-patient and In-patient Medical and Surgical services in addition to specialised services which include diabetes and hypertension clinics.

### Study population

The study population involved people living with diabetes who were at least 18years and visited the diabetes clinic in CCTH between January and February 2020

### Inclusion criteria

1. Type 1 and type 2 diabetes patients aged 18years and above
2. A physician clinically diagnosed the patient as having diabetes and receiving treatment during the research period.
3. Patient clinically diagnosed and on anti-glycaemic medication for at least six months.

### Exclusion criteria

1. Diabetes patients who were below the age of 18years
2. Individuals with acutely life-threatening conditions such as coma or mental impairment which may limit their cognitive ability to participate
3. Individuals who had been clinically diagnosed and on anti-glycaemic medication for less than six months

### Sample size determination

Data was collected from 250 participants living with diabetes aged at least 18 years. This was computed with a target population of 2800 (records obtained from CCTH diabetes clinic), a prevalence rate of diabetes among adult population of 6.46%,^18^ a 95% confidence interval with a 3% margin of error and a provision of 5% contingency. The sample size was determined using the Cochran equation for infinite population.^19^

### Sampling procedure

Although the target population was known, information about their places of residence and telephone numbers were inadequate. Therefore, the study employed consecutive (total enumerative) sampling to ensure that those who met the inclusion criteria were included. This was done within the study period, 1^st^ January to 29^th^ February 2020, until the required sample size of 250 was obtained.

### Data collection tools and procedure

A structured paper questionnaire was administered to the respondents to elicit responses on: their baseline (socio-demographic) characteristics; profile of diabetes; presence of co-morbidities; their level of anti-glycaemic medication-taking; patient-related factors; healthcare provider factors; and other factors that influence anti-glycaemic medication-taking. The ABC taxonomy tool validated in the African setting^20^,^21^ was used to measure anti-glycaemic medication-taking by operationalising it into four major domains with 8-items.^5^,^8^ The four major domains were “Taking medications appropriately according to the instructions of healthcare professionals (3-items)”, “filling prescriptions (1-item)”, “Practicing behavioural modifications (3-items)” and “Showing up for follow-up appointments (1-item)”.^5^ Taking medications appropriately was defined and scored as follows: initiation (when the patient takes the first dose of a prescribed medication- a score of 0 and 1 otherwise); implementation (the extent to which a patient's actual dosing corresponds to the prescribed dosing regimen, from initiation until the last dose is taken -a score of 0 and 1 otherwise) and discontinuation (which marks the end of therapy-a score of 0 and 1 otherwise)^5^. Practising behavioural modifications was also defined and scored as follows: an organised, multi-component diabetes-specific programme with repeated interactions by one or more trained individuals, with a duration of ≥4 weeks, to improve disease control and/or patient health outcomes, and consisting of (a) Diabetes self-management education and support (DSME)- a score of 0 and 1 otherwise; (b) a structured dietary intervention (related to any of weight loss, glycaemic control, or reducing risk for complications) -a score of 0 and 1 otherwise; and (c) a structured exercise or physical activity-a score of 0 and 1 otherwise^22^. Filling prescriptions after the discontinuation phase and showing up for follow-up appointments as scheduled were each scored- 0 and 1 otherwise.

### Data management

Data were entered, coded and stored on a personal laptop with a password. Data were double-entered using Microsoft Access software (2016 version). Verification checks were applied and discrepancies in records corrected using the completed questionnaires. Study participants were given unique codes to avoid duplicate records.

### Data analysis

The cleaned data were exported to Stata version 14.0 for statistical analysis. The total score for anti-glycaemic medication-taking of each study participant on the operationalised ABC taxonomy tool was calculated. The distribution of participants' total scores was skewed with a median of 1. Therefore, participants with total scores below one was coded as having high anti-glycaemic medication-taking scores and scores of one or above as having low anti-glycaemic medication-taking scores. The coefficient of reliability, Cronbach's α for the medication-taking scale was 0.7. Socio-demographic characteristics of respondents were described using frequencies and percentages. Socio-demographic, clinical and other factors associated with the main outcome, medication-taking (whether high or low) was determined using chi-square and Fisher's exact tests (where indicated). All statistically significant variables were used to construct univariate and multivariate logistic regressions. However, age and BMI of respondents were considered as a priori variables. Hence, they were considered in the regression models irrespective of being statistically significant or not. The main outcome for the logistic regressions was anti-glycaemic medication-taking. All tests were two-tailed and p<0.05 was considered statistically significant.

### Ethical considerations

Ethical clearance was acquired from the Institutional Review Board, University of Coast and Cape Coast Teaching Hospital Ethical Review Committee with the protocol identification numbers UCCIRB/CHAS/2019/123 and CCTHERC/EC/2020/005, respectively. Informed consent was sought from each respondent before administering the questionnaires. For non-English speakers and those without formal education, consent was sought in the local dialect. A consent form was administered, which explained the purpose of the research and why the participants were being recruited for the study. All respondents gave their consent by signing or thumb-printing the consent form. Personal identifiers of the participants were not recorded to ensure confidentiality.

## Results

Table 1 presents the socio-demographic characteristics of diabetes mellitus patients. The majority of the respondents were females (68.4%). More than half of the respondents were married (58 %) and were 60 years or above (58 %).

**Table 1:** Socio-demographic characteristics of respondents

Variable	n (%)
Sex	
**Male**	79 (31.6)
**Female**	171 (68.4)
Age	
**18–39**	9 (3.6)
**40–59**	96 (38.4)
**≥60**	145 (58.0)
Educational level	
**No formal education**	56 (22.4)
**Primary**	32 (12.8)
**Midd/JSS/JHS**	93 (37.2)
**Secondary**	25 (10.0)
**Tertiary**	44 (17.6)
Marital status	
**Single**	6 (2.4)
**Married**	145 (58.0)
**Divorced**	35 (14.0)
**Widow/Widower**	64 (25.6)
Occupation	
**Unemployed**	100 (40.0)
**Farming/Fishing**	33 (13.2)
**Trading**	48 (19.2)
**Artisan**	11 (4.4)
**Civil/Public Servant**	31 (12.4)
**Food related work**	27 (10.8)
Religion	
**Christianity**	225 (90.0)
**Islam**	25 (10.0)

As shown in Table 2, 105 (42%) had high anti-glycaemic medication-taking scores and 145 (58 %) had low medication taking scores. Within the high medication- taking category, greater proportions of respondents had had tertiary education (58.1%), were aged 60 years and above (47.6%), were unemployed, were single (50%), and Christian.

**Table 2:** Socio-demographic characteristics and anti-glycaemic medication-taking

Variable	Low Medication Taking	High Medication Taking	p-value
	n (%)	n (%)	
Sex			
**Male**	46 (58.2)	33 (41.8)	0.960
**Female**	99 (57.9)	72 (42.1)	
Age			
**18–39**	6 (66.7)	3 (33.3)	0.113
**40–59**	63 (65.6)	33 (34.4)	
**≥60**	76 (52.4)	69 (47.6)	
Educational level			
**No formal education**	36 (65.5)	19 (34.5)	0.116
**Primary**	21 (65.6)	11 (34.4)	
**Midd/JSS/JHS**	57 (61.3)	36 (38.7)	
**Secondary**	13 (52.0)	12 (48.0)	
**Tertiary**	18 (41.9)	25 (58.1)	
Marital status			
**Single**	3 (50.0)	3 (50.0)	0.764
**Married**	82 (56.6)	63 (43.5)	
**Divorced**	23 (65.7)	12 (34.3)	
**Widow/Widower**	37 (57.8)	27 (42.2)	
Occupation			
**Unemployed**	51 (51.0)	49 (49.0)	0.072
**Farming/Fishing**	19 (57.6)	14 (42.4)	
**Trading**	28 (58.3)	20 (41.7)	
**Artisan**	10 (90.9)	1 (9.1)	
**Civil/Public Servant**	17 (54.8)	14 (45.2)	
**Food related work**	20 (74.1)	7 (25.9)	
Religion			
**Christianity**	127 (56.4)	98 (43.6)	0.135
**Islam**	18 (72.0)	7 (28.7)	

None of the socio-demographic variables was significantly associated with anti-glycaemic medication-taking.

Table 3 presents the clinical and other factors associated with medication taking. Factors found to be significantly associated with medication taking included lack of finance (p<0.001), medication interfering with meal plan (p<0.001), presence of comorbidities (p=0.017), and poor family support (p=0.015).

**Table 3:** Clinical and other factors associated with anti-glycaemic medication-taking

Variable	Low Medication- Taking	High Medication -Taking	p-value (*p<0.05)
	n (%)	n (%)	
BMI			
**Underweight**	1 (33.3)	2 (66.7)	0.627
**Normal**	41 (63.1)	24 (36.9)	
**Overweight**	44 (55.0)	36 (45.0)	
**Obese**	54 (58.7)	38 (41.3)	
Age at onset of disease			
**<30**	6 (66.7)	3 (33.3)	0.374
**30–39**	15 (62.5)	9 (37.5)	
**40–59**	95 (60.5)	62 (39.5)	
**≥60**	29 (48.3)	31 (51.7)	
Duration of disease (years)			
**<1**	8 (53.3)	7 (46.7)	0.227
**1–5**	52 (55.9)	41 (44.1)	
**6–10**	43 (68.3)	20 (31.7)	
**11–20**	32 (58.2)	23 (41.8)	
**>20**	10 (41.7)	14 (58.3)	
Family history			
**No**	49 (56.3)	38 (43.7)	0.097
**Yes**	84 (56.4)	65 (43.6)	
**Don't know**	12 (85.7)	2 (14.3)	
Presence of co-morbidities			
**No**	49 (70.0)	21 (30.0)	0.017*
**Yes**	96 (53.3)	84 (46.6)	
Lack of finance			
**No**	93 (49.0)	97 (51.1)	<0.001*
**Yes**	52 (86.7)	8 (13.3)	
Medication interferes with meal plan			
**No**	101 (50.5)	99 (49.5)	<0.001*
**Yes**	44 (88.0)	6 (12.0)	
Forgetfulness			
**No**	50 (33.1)	101 (66.9)	<0.001*
**Yes**	95 (96.0)	4 (4.0)	
Side effects			
**No**	130 (56.0)	102 (44.0)	0.024*
**Yes**	15 (83.3)	3 (16.7)	
Perceived feelings of high dose			
**No**	137 (57.1)	103 (42.9)	0.199
**Yes**	8 (80.0)	2 (20.0)	
Poor family support			
**No**	134 (56.3)	104 (43.7)	0.015*
**Yes**	11 (91.7)	1 (8.3)	
Regular monitoring of blood glucose			
**No**	93 (62.0)	57 (38.0)	0.117
**Yes**	52 (52.0)	48 (48.0)	
Own modification of prescribed dose			
**No**	125 (55.8)	99 (44.2)	0.039*
**Yes**	20 (76.9)	6 (23.1)	
Own modification in the timing of medication			
**No**	80 (50.3)	79 (49.7)	0.001*
**Yes**	65 (71.4)	26 (28.6)	
Good knowledge about prescribed medication			
**No**	94 (65.3)	50 (34.7)	0.007*
**Yes**	51 (48.1)	55 (51.9)	
Involved in treatment decisions			
**No**	6 (31.6)	13 (68.4)	0.015*
**Yes**	139 (60.2)	92 (39.8)	

Additionally, side effects of drugs (p=0.024), forgetfulness in taking medication (p<0.001), modification of prescribed dose of medication (p=0.039), modification of timing of medication (p=0.001), good knowledge about prescribed medication (p=0.007) and involvement in treatment decisions (p=0.015) were associated with medication taking. Table 4 presents the univariate and multivariate logistic regressions depicting the unadjusted (OR) and adjusted (aOR) odds ratios of factors that influence anti-glycaemic medication-taking.

**Table 4:** Predictors of anti-glycaemic medication-taking

Variable	Univariate Model		Multivariate Model	
	OR (95% CI)	p-value	aOR (95% CI)	p-value
Age				
**18–39**	1		1	
**40–59**	1.05 (0.25–4.36)	0.950	2.06 (0.23–18.14)	0.517
**≥ 60**	1.82 (0.44–7.54)	0.412	2.44 (0.29–20.73)	0.415
BMI				
**Underweight**	1		1	
**Normal**	0.29 (0.03–3.40)	0.326	0.09 (0.00–10.58)	0.326
**Overweight**	0.41 (0.04–4.70)	0.473	0.15 (0.00–17.61)	0.435
**Obese**	0.35 (0.03–4.02)	0.401	0.11 (0.00–12.17)	0.353
Have good knowledge about their medication				
**No**	1		1	
**Yes**	2.03 (1.21–3.39)	0.007*	2.34 (1.10–4.98)	0.028*
Presence of co-morbidities				
**No**	1		1	
**Yes**	2.04 (1.13–3.68)	0.018*	1.68 (0.64–4.43)	0.296
Lack of finance				
**No**	1		1	
**Yes**	0.15 (0.07–0.33)	<0.001*	1.12 (0.31–3.98)	0.864
Interferes with meal plan				
**No**	1		1	
**Yes**	0.14 (0.06–0.34)	<0.001*	1.26 (0.27–5.76)	0.769
Forgetfulness				
**No**	1		1	
**Yes**	0.02 (0.01–0.06)	<0.001*	0.02 (0.00–0.64)	<0.001*
Side effects				
**No**	1		1	
**Yes**	0.25 (0.07–0.90)	0.034*	0.33 (0.06–1.74)	0.193
Poor family support				
**No**	1		1	
**Yes**	0.12 (0.01–0.92)	0.042*	0.66 (0.05–8.35)	0.750
Involvement in treatment plan				
**No**	1		1	
**Yes**	0.31 (0.11–0.83)	0.020*	0.12 (0.02–0.64)	0.014*
Modification in the prescribed dose				
**No**	1		1	
**Yes**	0.38 (0.15–0.98)	0.045*	0.63 (0.15–2.70)	0.535
Modification in the timing of medication				
**No**	1		1	
**Yes**	0.41 (0.23–0.70)	0.001*	0.98 (0.42–2.32)	0.971

* p<0.05

In the multivariate regression, patients' forgetfulness, good knowledge about medication and involvement in treatment plans were the only statistically significant factors. Patients with good knowledge about medication were 2.34 times more likely to have high anti-glycaemic medication-taking compared with patients with poor knowledge (aOR=2.34, 95% CI: 1.10–4.98, p=0.028). Patients who were involved in the treatment plan were 88% less likely to have high anti-glycaemic medication-taking as compared to those who were not involved in the treatment plan (aOR=0.12, 95% CI: 0.02–0.64, p=0.014). In addition, patients with forgetfulness had 98% less likelihood of having high anti-glycaemic medication-taking compared to those who were not forgetful (aOR=0.02, 95% CI: 0.00–0.64, p<0.001).

## Discussion

Diabetes mellitus is managed both non-pharmacologically and pharmacologically. To achieve the desired goals of treatment, patients need to be stringent in adhering to their prescribed drug therapy and lifestyle modification. However, research has shown that most individuals do not take the medications as prescribed by their physician due to individual peculiarities. The current study was therefore conducted among diabetics to assess patient's self-reported medication-taking. It is to be noted that, adherence and non-adherence used in previous studies have been reclassified as high and low medication-taking respectively in this study, in order to avoid stigma. The prevalence of high anti-glycaemic medication-taking in this study was 42% (105 out of 250) whilst that of low was (58%). An almost similar result was obtained in Cameroon, where Aminde *et al* showed that medication- taking was low among 54.4% of their participants.^7^The finding in this study that medication taking was low amongst a greater proportion of the respondents can be explained by the fact that most respondents were resident in rural areas and got little information about their disease and medication except that obtained from their healthcare providers. . They are further exposed to false information by some local herbal doctors and un-informed neighbours in their communities, leading to the low rates of medication-taking observed. Rates of medication-taking were however much lower in Uganda (16.7%) and Nigeria (27.5%), respectively.^7^

Estimates from WHO, indicate that about half (≈50%) of patients living with chronic diseases in developed countries followed treatment recommendations by their healthcare providers, with lower rates for people living with diabetes.^8^ On the contrary, one study conducted in Ethiopia and two in India showed that patients' self-reported anti-glycaemic medication-taking rates were 72.2%, 66.9% and 57.5%, respectively.^23^,^24^,^25^ These were relatively higher than the rate obtained in this study. Variations in the healthcare services, socio-economic status and metrics used for assessment of medication-taking across the study settings could account for the differences in the observed levels.

A systematic review by Krueger and colleagues showed important relationships between age and medication taking in seven of the papers reviewed.^26^This study revealed that as age increased, anti-glycaemic medication-taking increased. The rate of medication-taking among those 60 years and over was 47.6% whilst the rate for those below 40 years was 33.3%. This finding is consistent with those of other studies where increasing age was correlated with higher medication-taking rate.^23^,^26^In this present study, rate of medication-taking was similar in both genders. Also, the highest medication-taking rate was noted among patients with tertiary education (58.1%), followed by those who had completed senior secondary school (48%), and those who completed junior secondary school (38.7%). These findings are consistent with studies in United Arab Emirates (UAE).^10^

As reported by Belayneh et al., the longer the duration of diabetes, the lower the medication-taking rate among people living with diabetes.^23^ However, this was not so in the present study as respondents with the longest duration of diabetes (>20years) recorded the highest medication-taking rate (58.3%). These findings contrasted the results of two other studies, which indicated a negative relationship between the duration of diabetes and patient's anti-glycaemic medication-taking rate.^10^,^24^

According to Labrador et al., the majority of patients considered having knowledge about their disease and medication as a relevant component of good medication taking.^27^ Research carried out by Atinga and colleagues in Ghana, demonstrated that knowledge about medications had a statistically significant association with, but negatively influenced medication-taking.^28^ Although this study also showed significant relationship between knowledge and medication taking, knowledge positively influenced it. Involving patients in management of their diseases is key in ensuring compliance to treatment. Recent evidence provided by Labrador et al., indicated that involving people living with type 2 diabetes in the management of their disease improved medication-taking.^27^ In the same study, it was stated that if patients realised that their preferences (with respect to the management of their disease) were considered, they became motivated and committed, increasing their medication-taking rate and improving clinical outcomes.^27^ However, it was revealed in this study that patients who were involved in their treatment plan were 88% less likely to adhere to their medication. It is possible that factors such as patients' preferences in management, as revealed by Labrador and colleagues, were not considered during involvement in management. This finding will need further investigation.

Forgetfulness has been identified as one of the major contributors to the low anti-glycaemic medication-taking rate in several studies.^12^,^18^,^29^ In this study, forgetfulness was found to be significantly associated with medication-taking.

If one was forgetful about taking medications, he or she was 98% less likely to have high medication-taking rate. Having trouble remembering to take medication is a common concern. Forgetting to take medication can be related to how many times a day a medication is prescribed. For some other people, forgetting to take medications is due to the medication not being part of a routine.

This study had some limitations that were likely to affect the study's conclusions. First of all, the hospital-based consecutive (total enumerative) sampling could introduce bias in the sampling of participants. Secondly, the study considered all adults living with diabetes irrespective of the type and treatment regimen the participants were on. Thirdly, the type of comorbidities and the possibility of polypharmacy, especially among the elderly, could influence medication-taking but was not analysed in this study.

## Conclusion

This study revealed that the level of anti-glycaemic medication-taking was 42% among patients attending the diabetes clinic at CCTH. Furthermore, good knowledge about one's medication, forgetfulness and patient involvement in treatment decisions were predictors of anti-glycaemic medication-taking in patients attending the diabetes clinic at CCTH.
